# Genetic Relationships among Tall Coconut Palm (*Cocos nucifera* L.) Accessions of the International Coconut Genebank for Latin America and the Caribbean (ICG-LAC), Evaluated Using Microsatellite Markers (SSRs)

**DOI:** 10.1371/journal.pone.0151309

**Published:** 2016-03-14

**Authors:** Carina Mendes Loiola, Alinne Oliveira Nunes Azevedo, Leandro E. C. Diniz, Wilson Menezes Aragão, Carlos Diego de O. Azevedo, Pedro Henrique A. D. Santos, Helaine Christine C. Ramos, Messias Gonzaga Pereira, Semíramis R. Ramalho Ramos

**Affiliations:** 1 Federal Rural University of Semi-Árido/Embrapa Coastal Tablelands, Aracaju, Sergipe, Brazil; 2 North Fluminense State University (UENF), Campos dos Goytacazes, Rio de Janeiro, Brazil; 3 Embrapa Coastal Tablelands, Aracaju, Sergipe, Brazil; Washington University, UNITED STATES

## Abstract

The diversity and genetic relationships among two accessions of tall coconut palms collected in Brazil and seven accessions introduced from different geographic regions of the world were analyzed using 25 microsatellite primers, 19 of which were polymorphic and detected between 4 and 10 alleles per locus, with an average of 6.57. The observed and expected heterozygosity ranged from 0.25 and 0.40 in the Rennell Islands Tall (RIT) accession to 0.54 and 0.62 in the Polynesian Tall (PYT) accession. The analysis of genetic structure resulted in the formation of five distinct groups. The first group was formed by the accessions Brazilian Tall—Praia do Forte (BRTPF), Brazilian Tall—Merepe (BRTMe) and West African Tall (WAT); the second group consisted of Malaysian Tall (MLT); the third group of RIT; the fourth group of Vanuatu Tall (VTT); and the fifth group of Rotuman Tall (RTMT), Tonga Tall (TONT) and PYT. The dendrogram based on the nearest-neighbor method detected the formation of two main groups and five subgroups, indicating that the genetic relationships of the accessions are based on their geographic regions of origin. The analyses revealed genetic relationships between the accessions collected in Brazil and the accession from Africa, and among palms from South East Asia and the South Pacific, confirming the common origin of these accessions. The information obtained in this study can guide decisions on germplasm conservation activities and the efficient selection of genetically divergent parents for use in coconut breeding programs in Brazil, which are attempting to select for disease resistance, mainly to lethal yellowing, among other characteristics.

## Introduction

The coconut palm (*Cocos nucifera* L.) has played an important role in the mobility of humans as source of food and water. Its evolution, dissemination and classification can be considered as a logical sequence of spread by floating, selection by cultivation, hybridization and introgression of the two contrasting types—representing two important gene sets (Niu Kafa and Niu Vai)—which resulted in its wide range of varieties and a pantropical distribution [1, 2].

Based on analyses of the genetic divergence using DNA markers, a classification of coconut palms into two types was proposed, according to different geographical regions: i.e. 1-the Pacific Group and 2-the Indo-Atlantic Group. This classification contributed for understanding better the dispersal and genetic diversity that exists among populations of coconut palm [3, 4]. From India, Europeans introduced the coconut palm to the Atlantic Coast of Africa, South America and the Caribbean [5]. However, there is no consensus about the period when coconuts were introduced in the Pacific Coast of Latin America. Some authors suggests that it occurred during the Spanish conquest [6] while others says that coconuts were already present in the region in pre Colombian period [7].

The coconut served the Portuguese on their overseas expeditions and the introduction in Brazil was a consequence. According to historical accounts, the coconut palms were initially introduced in 1553 in the coast of Bahia, Brazil [8]. Mozambique is assumed to be the common source and the Cape Verde Islands a route of dissemination of coconuts [9]. The common tall variety of coconut seen in the state of Bahia appears to be similar to other varieties seen in Jamaica, South America and West and East Africa [9] as well as with other varieties belonging to the typical Indo-Atlantic group [10]. Other introductions probably occurred between the XV and XIX centuries [9], when the Cape Verde Islands served as a trading port between Portugal, Brazil and Africa. However, no information has been found about these potential introductions, to Brazil, like was done by Zizumbo-Villareal in Mexico [11]. In the 1980s, other coconut palms were introduced by both private and public companies, which subsidized research activities on genetic resources and breeding [12]. These actions began with the introduction by the Brazilian Agricultural Research Corporation [Empresa Brasileira de Pesquisa Agropecuária (EMBRAPA)] of seven accessions of Tall Coconut Palms from the International Coconut Genebank for Africa and the Indian Ocean (ICG-AIO), located at the Centre National de Recherche Agronomique (CNRA), Côte d'Ivoire, which were subsequently deposited in the Active Germplasm Bank at EMBRAPA in the State of Sergipe, Brazil. EMBRAPA also performed exploration and collection of tall coconut palm accessions in the northeast region of Brazil. The existing populations in this region have been established for more than 450 years. However, there is still little available information about their structure and genetic diversity.

In 2006, the Germplasm Bank of Brazil was associated with the International Coconut Genetic Resources Network (COGENT) and was renamed the International Coconut Genebank for Latin America and the Caribbean (ICG-LAC). One of the many objectives of ICG-LAC is to be the host location for conserving of the genetic variability of the species in this region. There are 29 accessions of dwarf and tall coconut palms conserved at ICG-LAC, and despite the small number of accessions, it is the second most important genebank in the Americas, after that of Jamaica (http://www.cogentnetwork.org/conserved-germplasm-catalogue). Different characterization and evaluation activities have been conducted at the genebank, and the Brazilian Tall—Praia do Forte and Brazilian Tall—Merepe populations have shown to be superior in terms of their fruit components, primarily regarding fruit weight, solid albumen and coconut water volume [13]. These characteristics make the use of these accessions in the coconut agroindustry in Brazil very promising due to the high demand for coconut water in the Brazilian marketplace. Notably, Brazilian Tall—Praia do Forte is already being used in the country by both public and private institutions as the male parent in the production of intervarietal hybrids with yellow dwarf, green dwarf and red dwarf varieties [14].

Investigations of genetic divergence examining the populations surveyed in Brazil have been performed using morphological characteristics of the fruits, resulting in the identification of divergent populations [15]. Studies have also been carried out using microsatellites markers (SSRs), which have identified promising germplasm sources for cultivar breeding programs in Brazil [16]. Populations of Brazilian Tall—Praia do Forte and Merepe—were shown to be genetically distinct from each other [16] but similar to West African Tall by RAPD markers [17]. However, because this type of molecular marker is dominant and non-reproducible, it is difficult to obtain accurate and consistent genotype information for genetic studies. Therefore, in order to better estimate the divergence and genetic relationships between the collected accessions and those that were introduced into Brazil, molecular techniques that are more informative and reliable need to be used. These techniques allow one to infer possible relationships between varieties of coconut palms that show some resistance to lethal yellowing disease, which has been destroying thousands of hectares of coconut palms in Africa, North America and the Caribbean [18]. The aim of this study was to evaluate the levels of diversity and the genetic relationships among two accessions of tall coconut palms collected in Brazil and seven accessions of coconut palms introduced from different geographical regions of the world.

## Materials and Methods

The accessions evaluated in this study are preserved at ICG-LAC, located in the Experimental Station of Betume, municipality of Neópolis, Sergipe state, Brazil (Table 1). Ten individuals were randomly selected from each accession for the molecular analysis. This number of individuals per accession was suggested as being sufficient to genetically characterize populations of distinct origin with accuracy [19].

**Table 1:** Discrimination, international code, origin, geographic region of the nine tall coconut palm accessions, preserved in the International Coconut Genebank for Latin America and the Caribbean (ICG-LAC).

Accessions	Code	Origin^a^	Geographic Region ^b^
Brazilian Tall Praia do Forte	BRTPF	Brazil	South America
Brazilian Tall Merepe	BRTMe	Brazil	South America
West African Tall	WAT	Côte d’Ivoire	West Africa
Rennell Islands Tall	RIT	Solomon	South Pacific
Polynesian Tall	PYT	Tahiti	South Pacific
Rotuman Tall	RTMT	Fiji	South Pacific
Tonga Tall	TONT	Tonga	South Pacific
Vanuatu Tall	VTT	Vanuatu	South Pacific
Malayan Tall	MLT	Malaysia	Southeast Asia

^a^ Country of origin of the collection or the accessions conservation site

^b^ Geographic region of the country of origin of the accessions

### DNA Extraction and Analysis with SSR Markers

DNA was extracted from 500 mg of leaflets of the first leaf using the CTAB Protocol [20] with some modifications in the volume of the reagents used (700 μL of chloroform-isoamyl alcohol (24:1), 500 μL of cold isopropyl alcohol, 450 μL of pure ethyl alcohol and 300 μL of 70% ethyl alcohol) and was quantified using a NanoDrop^®^ 2000c spectrophotometer (Thermo Scientific^®^). The SSR marker analyses were performed as previously described [19]. The PCR products were analyzed using a fluorescence-based capillary electrophoresis system, the AdvanCE^™^ FS96 system (Advanced Analytical Software), with DNF900 gel kit, for 120 minutes, at a current of 7.50 Kw to separate the DNA fragments, with a 5 pg/μL sensitivity and a 5 bp resolution. For this analysis, 1 μL of each amplification product was diluted in 79 μl of ultrapure water and placed in 96-well microplates. A 50-bp molecular weight marker (Invitrogen USA) was included in the last well of the microplate to determine the size of the amplified fragments.

### Statistical analysis

The program GENALEX 6.3 [21] was employed to estimate the number of alleles, number of effective alleles, frequency of the main allele, expected heterozygosity (H_E_) and observed heterozygosity (H_O_) and Shannon index

$$\left(H\right)=\;-\overset{s}{\underset{i=1}{\sum }}p\;i\;\text{ln}\;p_{i}$$

The coefficient of inbreeding (F = (H_E_−H_O_)/H_E_ = 1−(H_O_ /H_E_)) and polymorphism information content (PIC) were calculated with PowerMarker version 3.25 [22]

$$PIC=1-\overset{l}{\underset{i=l}{\sum }}p_{\begin{array}{c} 1 \end{array}}^{2}-\;\overset{l=1}{\underset{i=1}{\sum }}\overset{l}{\underset{j=i+1}{\sum }}2P_{i}^{2}\;P_{j}^{2}$$

The Pearson´s Chi-square test [23] was applied to H_E_ and H_O_ to determine whether genotype frequencies follow Hardy-Weinberg equilibrium. To analyze the genetic structure of the population, Bayesian clustering methods in the program STRUCTURE 2.3.1 were used [24], where the number of groups (K) was determined using the ΔK method [25]. The admixture model and correlated allele frequency were done for 20 runs for each K value, which ranged from 1 to 9 (10,000 burn-in, 1,000,000 permutations).

Roger’s dissimilarity coefficient [26] was calculated with the aid of the program GENALEX 6.3 [21]. Cluster analysis of the accessions was performed based on the nearest-neighbor method [27], using the program MEGA version 5 [28]. A Bootstrap analysis was also performed to assess the degree of confidence of each node in the dendrogram. The two analyses were conducted using the program R [29], with the "cluster" and "Rcmdr" packages.

## Results and Discussion

Twenty-five pairs of SSR primers were used to analyze 90 individuals of nine tall coconut palm accessions from different geographical regions, 19 of which showed polymorphic patterns, while six showed no amplification. A total of 125 alleles were identified with the 19 polymorphic SSRs (Table 2), which generated an average of 6.57 alleles/locus, ranging between 4 and 10 alleles. These results are in the same range of variation as those obtained in coconut palm belonging to ecotypes from different geographical regions in the world [30].

**Table 2:** Results for 19 microsatellite loci examined in nine tall coconut palm accessions from different geographic regions conserved in the International Coconut Genebank for Latin America and the Caribbean (ICG-LAC).

Primers	Number of alleles found	Effective number of alleles (N_e_)	Frequency of the main allele	PIC
CnCir A3	8	2.44	0.24	0.80
CnCir B12	8	3.31	0.25	0.79
CnCir C12	4	2.35	0.40	0.63
CnCir E2	8	2.59	0.38	0.76
CnCir E10	5	2.25	0.47	0.60
CnCir E12	8	3.52	0.31	0.78
CNZ40	7	2.48	0.38	0.74
CAC02	9	3.88	0.28	0.80
CNZ10	10	2.85	0.36	0.75
CNZ43	9	2.31	0.20	0.82
CNZ44	8	2.69	0.30	0.80
CnCir B3	7	2.79	0.34	0.73
CNZ 01	5	1.89	0.37	0.72
CNZ02	5	2.76	0.32	0.74
CAC21	4	2.06	0.46	0.63
CAC23	4	1.97	0.41	0.59
CAC71	4	1.35	0.55	0.44
CAC84	5	1.53	0.48	0.60
CAC50	7	2.87	0.23	0.80
Total	125			
Average	6.57	2.52	0.35	0.71

The accessions were evaluated using the Shannon index, which has been applied specifically in genetic studies as a measure of diversity within the population and resembles a genotypic richness index [31] (Table 3). These results demonstrate the wide diversity or genotypic richness of these accessions preserved in the genebank (ICG-LAC). Heterozygosity estimates for the accessions indicated that the expected values (He) were somewhat larger than the observed values (Ho). The estimates for Ho ranged from 0.25 (RIT) to 0.54 (PYT). The values of this parameter were slightly higher for the accessions from the South Pacific and Southeast Asia, consistent with previously published results [4]. Chi-square tests showed no significant differences between Ho and He for most of the accessions, indicating a predominance of panmixia, i.e., the occurrence of random crossings in the populations. Thus, most of the accessions are at Hardy-Weinberg equilibrium, and consequently, the coefficient of inbreeding (F) was not significantly different from 0 (Table 3). Only the results for the accessions BRTMe and WAT were significant. It is likely that different processes have occurred in these accessions that have resulted in substantial inbreeding. These inbreeding values were similar to those found in the WAT accession preserved at the Central Plantation Crops Research Institute (CPCRI) in India [32] and greater than the results observed in other Brazilian and African populations [16, 33].

**Table 3:** Genetic diversity of tall coconut palm accessions from different geographic regions conserved in the International Coconut Genebank for Latin America and the Caribbean (ICG-LAC). BRTPF = Brazilian Tall Praia do Forte, BRTMe = Brazilian Tall Merepe, TONT = Tonga Tall, RTMT = Rotuman Tall, MLT = Malayan Tall, RIT = Rennell Islands Tall, VTT = Vanuatu Tall, WAT = West African Tall and PYT = Polynesian Tall.

Accessions	Shannon Index	Observed heterozygosity (H_O_)	Expected heterozygosity (H_E_)	X^2^ ^a^ (d.f. = 8)	Inbreeding coefficient (F)
BRTPF	0.90	0.43	0.50	22.31	0.16
BRTMe	0.89	0.33	0.50	31.55*	0.34
TONT	1.08	0.50	0.60	13.21	0.17
RTMT	1.05	0.41	0.59	24.42	0.31
MLT	1.12	0.44	0.59	19.18	0.27
RIT	0.66	0.25	0.40	25.71	0.33
VTT	0.95	0.40	0.54	22.01	0.26
WAT	0.85	0.33	0.48	31.05*	0.32
PYT	1.13	0.54	0.62	12.71	0.12

^a^X^2^ calculated at the 5% probability level

**Fig 1:**
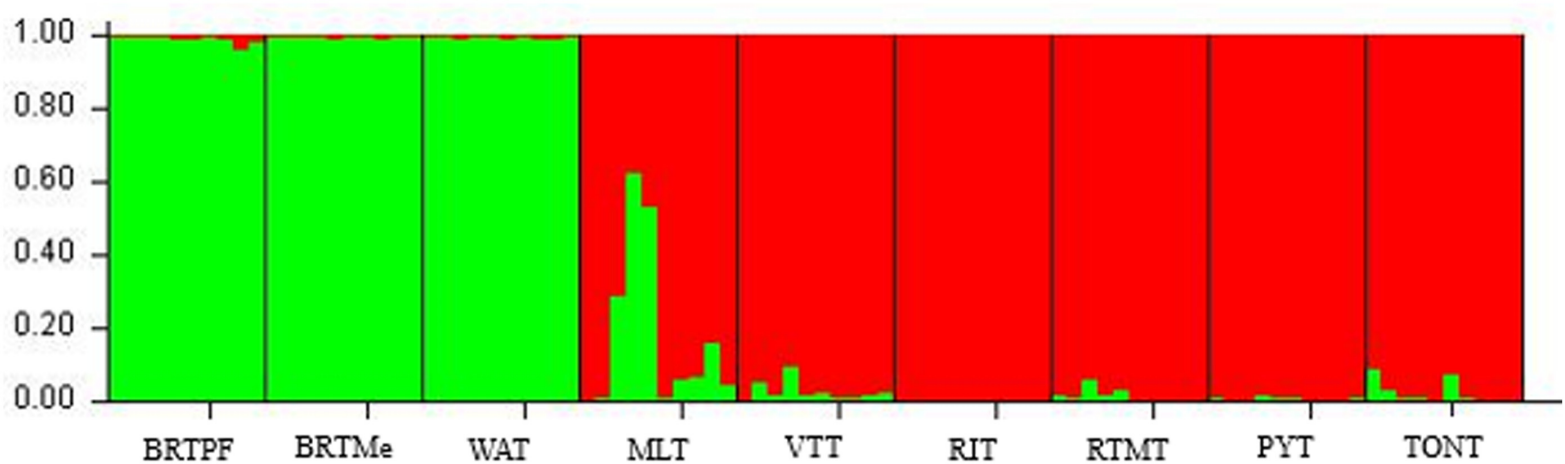
Results of the Structure analysis of a sample of 90 tall coconut palms. Population assignments for each accession are shown at K = 2 groups. BRTPF = Brazilian Tall Praia do Forte, BRTMe = Brazilian Tall Merepe, WAT = West African Tall, MLT = Malayan Tall, RIT = Rennell Islands Tall, VTT = Vanuatu Tall, RTMT = Rotuman Tall, TONT = Tonga Tall and PYT = Polynesian Tall.

The analysis of the genetic structure with Structure, indicated the formation of two different groups (K = 2) (Fig 1), which is consistent with previous studies with molecular markers [4, 34]. The first group was formed by the accessions collected in Brazil (BRTPF and BRTMe) and the accession from West Africa (WAT). The second group was formed by the accession of Southeast Asia (MLT) and the South Pacific (RIT, VTT, RTMT, TONT and PYT). Both combinations are in accordance with the genetic diversity classification proposed by the Generation Challenge Programme of the French Agricultural Research Centre for International Development (GCP/CIRAD) that divides the coconut into two genetically distinct groups. The first group covers the South Pacific, Southeast Asia and the Pacific coast of the Americas and is designated the A, or Pacific Group. The second group, which covers South Asia, Africa and the Atlantic coast of the Americas and the Caribbean, is designated the B or Indo-Atlantic Group [34].

**Fig 2:**
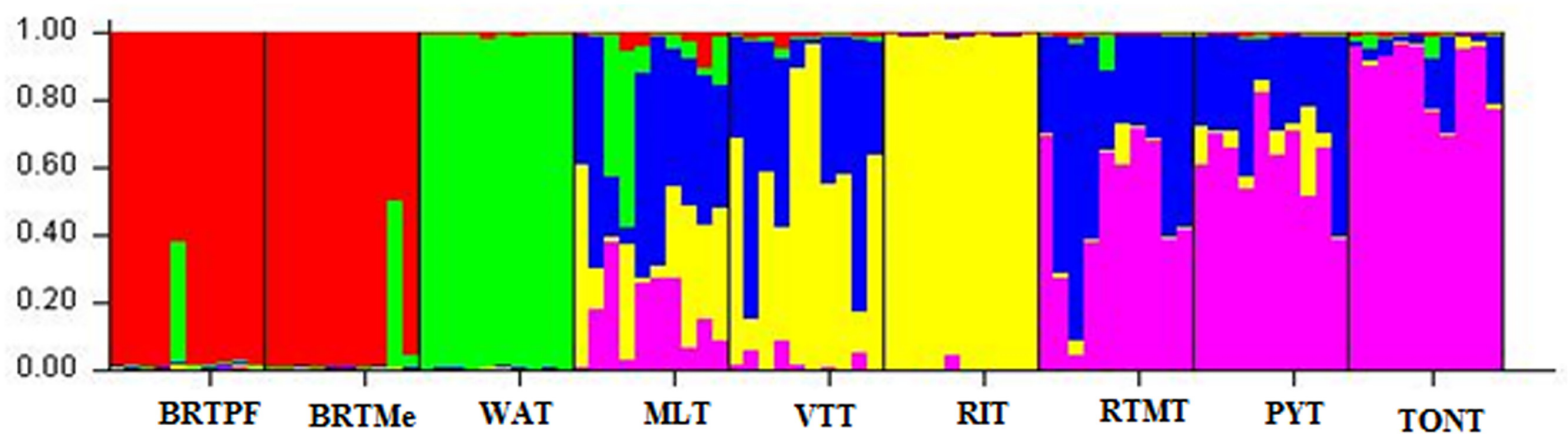
Results of the Structure analysis of a sample of 90 tall coconut palms. Population assignments for each accession are shown at K = 6 groups. Vertical black lines distinguish the groups. BRTPF = Brazilian Tall Praia do Forte, BRTMe = Brazilian Tall Merepe, WAT = West African Tall, MLT = Malayan Tall, RIT = Rennell Islands Tall, VTT = Vanuatu Tall, RTMT = Rotuman Tall, TONT = Tonga Tall and PYT = Polynesian Tall.

The presence of a secondary ΔK (S1 Fig) suggests substructure within the two main groups, one at K = 6 and another at K = 8. K = 6 (Fig 2) represents better the genetic relationships when compared with previous studies [4]. The first group was formed by the accessions collected in Brazil, and the second by WAT. The lack of allele sharing between these accessions and those introduced from South Pacific and Southeast Asia, show that these introductions did not involve admixed populations, such as found in eastern Africa and the western Indian Ocean [4].

The third group was strongly mixed and formed by MLT and VTT, while the fourth clear group was formed by the RIT accession. The fifth group was formed by RTMT and PYT, while the sixth with the TONT accession, both of them with high levels of allele sharing. These results follow patterns similar to those reported in Southeast Asia and Oceania by other authors [4], who also observed a mixture of subpopulations of coconut palms from the Indo-Atlantic and, the Pacific regions. This situation is likely due to the geographical proximity between this region and the eastern Indian Ocean and the trade established with South Asia. In Melanesia and Polynesia, the genetic composition is mostly similar to that of coconut palms from the South Pacific. This genetic heterogeneity within the accessions introduced from Southeast Asia and the South Pacific is correlated with the geographic dispersion of these populations.

**Fig 3:**
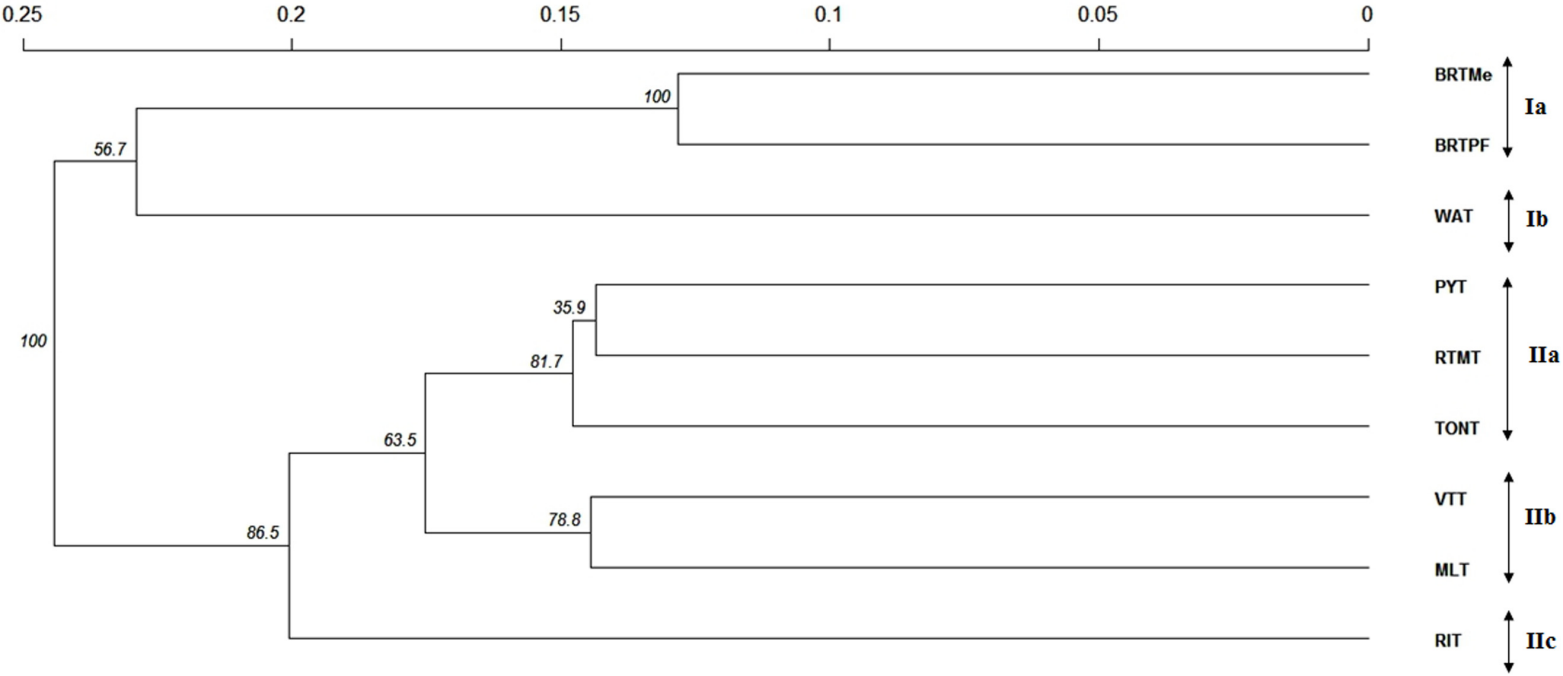
Dendrogram generated using the nearest-neighbor method, based on Roger’s standard genetic distance, for nine tall coconut accessions (Genetic distance = 0.39 and cophenetic correlation coefficient = 0.91). BRTPF = Brazilian Tall Praia do Forte, BRTMe = Brazilian Tall Merepe, WAT = West African Tall, MLT = Malayan Tall, RIT = Rennell Islands Tall, VTT = Vanuatu Tall, RTMT = Rotuman Tall, TONT = Tonga Tall and PYT = Polynesian Tall.

Cluster analysis detected the formation of two main groups (Fig 3). The first group was formed by coconut palms from Africa and South America and included two subgroups: I-a (BRTMe and BRTPF) and I-b (WAT). The second group, which contained the largest number of accessions, included the coconut accessions introduced from the South Pacific and Southeast Asia, subdivided into three subgroups: II-a (PYT, RTMT and TONT), II-b (VTT and MLT) and II-c (RIT). These two large groups, representing the Pacific and Indo-Atlantic, have been noted by other authors [4] and confirm the genetic relationships among accessions found in these geographical regions, which are due to the separate domestication events in the Indian Ocean and Pacific Ocean basins.

The genetic proximity between the accessions collected in Brazil and the WAT accession confirms the common origin of these accessions, as do the historical records that report that the tall coconut was introduced from the Cape Verde Islands. The strategic geographical location of the archipelago of Cape Verde in the Portuguese sea routes between Brazil and Africa contributed to these islands being used as a supply and trading outpost. The origin of these coconut palms would have been East Africa, India and Sri Lanka [1], when Vasco da Gama, returning from his travels to India, introduced seeds from Mozambique to the archipelago of Cape Verde [8]. This hypothesis is confirmed by the genetic similarity between the WAT, Mozambique Tall, Sri Lanka Tall and Andaman Tall (India) coconut palms [10, 35].

Other studies have confirmed genetic relationships between the Dominican Republic-Tall and the Mexican Atlantic Tall classified in the Indo-Atlantic Group, and populations of Brazilian Tall coconuts [18]. These findings indicate similarities in the history of the introduction of coconut palms to these three countries and the inclusion of accessions collected in Brazil in the Indo-Atlantic group.

In Brazil, populations of tall coconut were initially formed by a few closely related individuals, representing only a small fraction of the genetic variation in the source population. From the State of Bahia in the northeast region of Brazil, tall coconut expanded all along the northeast coast, which is a region that provides optimal soil and climate conditions for the development of this species, allowing this region to become the largest coconut producer in the country [36] and the location of the largest agroindustrial coconut companies [37]. The coconut palm has been growing in Brazil for almost five centuries, adapting to the various existing environmental conditions, suggesting that the observed gene structure is likely due to genetic drift, strongly influenced by the founder effect and human selection.

Within this scenario it is possible to suggest that some genetic variation exists between the materials introduced in Brazil five centuries ago and the WAT. In fact, this study detected significant differentiation between WAT and BRT accessions, both by the genetic structure analysis (Fig 3) as well as by the genetic distance (S1 Table). However, since WAT was described as an adapted landrace occurring from Côte d´Ivoire to Nigeria, through Ghana, Togo and Benin [38], there is not enough data to state that in Brazil there are new tall coconut ecotypes, since a single accession representing the West Africa was used in this study.

The populations collected in Brazil belong to the Indo-Atlantic group and this group proved to be susceptible to lethal yellowing (LY) [39], hence it is very likely that the populations collected in Brazil and preserved in the ICG-LAC are also susceptible. In the process of dissemination, LY has been diagnosed in African countries and has also reached coconut groves that surround the beaches of the Atlantic coast and some islands of North and Central America [40, 41]. However, the main threat of entry of this disease into Brazil comes from the Caribbean. Jamaica and Mexico have started trials to test for resistance, and in both countries the coconut palms in the Indo-Atlantic group were found to be highly susceptible to lethal yellowing [39]. In trials conducted in Jamaica, the Malayan Tall and Rotuma Tall, which belong to the Pacific group, showed lower mortality rates compared with other coconut palms from both the Indo-Atlantic group and the Pacific group [39]. This information may indicate that these accessions should be used for planting in new production areas as well as for inter- and intra-variety crossbreeding with Brazilian populations, which would serve as a preventive measure against the future entry of lethal yellowing in Brazil, since effective control methods have not yet been identified against this disease.

## Conclusions

The use of SSR markers increased the available information on the genetic relationships among the accessions collected in Brazil and those introduced from different geographic regions of the world that are preserved in the ICG-LAC. The accessions were assigned to the two main genetically distinct groups. The genetic relationships between accessions collected in Brazil and the African accession, as well as between the accessions of Southeast Asia and the South Pacific, agree with what is known about coconut in those regions. The information gathered in this study may guide decisions on germplasm conservation activities and the efficient selection of genetically divergent parents for use in coconut breeding programs in Brazil, that are attempting to select for resistance to lethal yellowing, among other characteristics.

## Supporting Information

S1 Fig Assessment of subpopulation number in Structure analysis. (TIF)

S2 Fig Results of the Structure analysis of a sample of 90 tall coconut palms. Population assignments for each accession are shown at K = 2 to K = 9 groups. BRTPF = Brazilian Tall Praia do Forte, BRTMe = Brazilian Tall Merepe, WAT = West African Tall, MLT = Malayan Tall, RIT = Rennell Islands Tall, VTT = Vanuatu Tall, RTMT = Rotuman Tall, TONT = Tonga Tall and PYT = Polynesian Tall.
(BMP)

S1 Table Genetic distance matrix among the nine tall coconut accessions using the Rogers’ distance. (DOCX)

S2 Table Allele frequencies for each locus. (DOCX)
