# Supplementary figures and images for: Genetic Relationships among Tall Coconut Palm (Cocos nucifera L.) Accessions of the International Coconut Genebank for Latin America and the Caribbean (ICG-LAC), Evaluated Using Microsatellite Markers (SSRs)
Source: PLoS One. 2016 Mar 14;11(3):e0151309. doi: 10.1371/journal.pone.0151309 (PMC4790901; doi:10.1371/journal.pone.0151309)

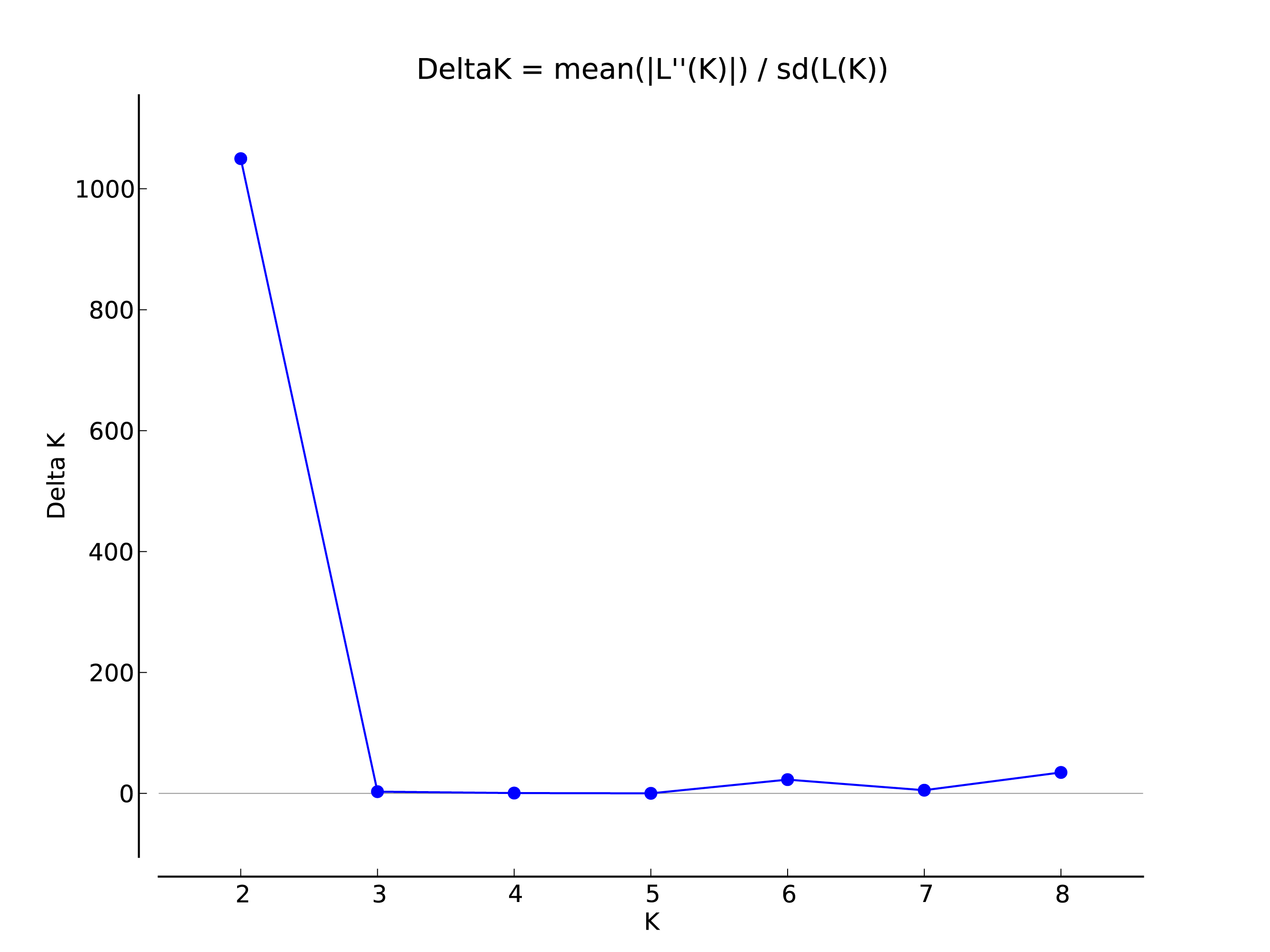

Supplement: S1 Fig — (TIF) [file pone.0151309.s001.tif]

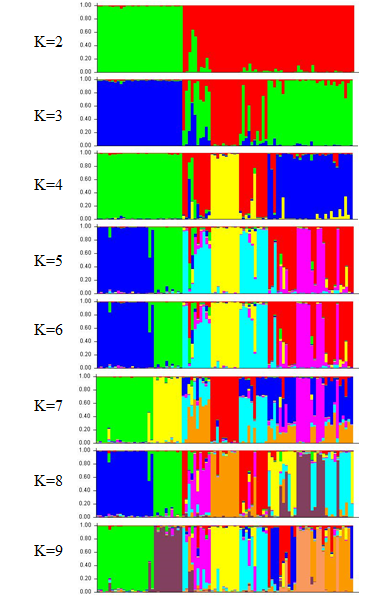

Supplement: S2 Fig — Population assignments for each accession are shown at K = 2 to K = 9 groups. BRTPF = Brazilian Tall Praia do Forte, BRTMe = Brazilian Tall Merepe, WAT = West African Tall, MLT = Malayan Tall, RIT = Rennell Islands Tall, VTT = Vanuatu Tall, RTMT = Rotuman Tall, TONT = Tonga Tall and PYT = Polynesian Tall. (BMP) [file pone.0151309.s002.bmp]
