# Supplementary material for: Genetic Relationships among Tall Coconut Palm (Cocos nucifera L.) Accessions of the International Coconut Genebank for Latin America and the Caribbean (ICG-LAC), Evaluated Using Microsatellite Markers (SSRs)
Source: PLoS One. 2016 Mar 14;11(3):e0151309. doi: 10.1371/journal.pone.0151309 (PMC4790901; doi:10.1371/journal.pone.0151309)
Supplement: S1 Table — (DOCX) [file pone.0151309.s003.docx]

| Tabela S1.Matriz de distância genética entre nove acessos de coqueiro gigante utilizando a distância de Rogers. | | | | | | | | | |
| --- | --- | --- | --- | --- | --- | --- | --- | --- | --- |
|  | BRTMe | BRTPF | MLT | WAT | PYT | RIT | RTMT | TONT | VTT |
| BRTMe | 0 | 0.26 | 0.46 | 0.47 | 0.50 | 0.61 | 0.50 | 0.50 | 0.48 |
| BRTPF | 0.26 | 0 | 0.46 | 0.50 | 0.48 | 0.57 | 0.49 | 0.48 | 0.47 |
| MLT | 0.46 | 0.46 | 0 | 0.45 | 0.32 | 0.40 | 0.34 | 0.34 | 0.29 |
| WAT | 0.47 | 0.50 | 0.45 | 0 | 0.54 | 0.61 | 0.52 | 0.54 | 0.51 |
| PYT | 0.50 | 0.48 | 0.32 | 0.54 | 0 | 0.38 | 0.29 | 0.29 | 0.37 |
| RIT | 0.61 | 0.57 | 0.40 | 0.61 | 0.38 | 0 | 0.43 | 0.45 | 0.35 |
| RTMT | 0.50 | 0.49 | 0.34 | 0.52 | 0.29 | 0.43 | 0 | 0.30 | 0.36 |
| TONT | 0.50 | 0.48 | 0.34 | 0.54 | 0.29 | 0.45 | 0.30 | 0 | 0.37 |
| VTT | 0.48 | 0.47 | 0.29 | 0.51 | 0.37 | 0.35 | 0.36 | 0.37 | 0 |
