# Supplementary material for: Genetic Relationships among Tall Coconut Palm (Cocos nucifera L.) Accessions of the International Coconut Genebank for Latin America and the Caribbean (ICG-LAC), Evaluated Using Microsatellite Markers (SSRs)
Source: PLoS One. 2016 Mar 14;11(3):e0151309. doi: 10.1371/journal.pone.0151309 (PMC4790901; doi:10.1371/journal.pone.0151309)
Supplement: S2 Table — (DOCX) [file pone.0151309.s004.docx]

**Table S2. Allele frequencies for each locus**

| **Locus** | **Allele/n** | **BRTPF** | **BRTME** | **TONT** | **RTMT** | **MLT** | **RIT** | **VTT** | **WAT** | **PYT** |
| --- | --- | --- | --- | --- | --- | --- | --- | --- | --- | --- |
| **CnCirA3** | N |  |  |  |  |  |  |  |  |  |
|  | **1** | 0,000 | 0,125 | 0,000 | 0,000 | 0,000 | 0,000 | 0,000 | 0,000 | 0,000 |
|  | **2** | 0,125 | 0,125 | 0,000 | 0,000 | 0,000 | 0,000 | 0,000 | 0,000 | 0,000 |
|  | **3** | 0,875 | 0,375 | 0,000 | 0,000 | 0,000 | 0,000 | 0,000 | 0,000 | 0,000 |
|  | **4** | 0,000 | 0,375 | 0,100 | 0,000 | 0,071 | 0,000 | 0,000 | 0,600 | 0,000 |
|  | **5** | 0,000 | 0,000 | 0,400 | 0,571 | 0,214 | 0,000 | 0,333 | 0,400 | 0,143 |
|  | **6** | 0,000 | 0,000 | 0,100 | 0,214 | 0,571 | 0,688 | 0,250 | 0,000 | 0,571 |
|  | **7** | 0,000 | 0,000 | 0,300 | 0,143 | 0,000 | 0,313 | 0,000 | 0,000 | 0,000 |
|  | **8** | 0,000 | 0,000 | 0,100 | 0,071 | 0,143 | 0,000 | 0,417 | 0,000 | 0,286 |
| **CnCirB12** | **N** |  |  |  |  |  |  |  |  |  |
|  | **1** | 0,000 | 0,000 | 0,150 | 0,150 | 0,000 | 0,000 | 0,167 | 0,000 | 0,100 |
|  | **2** | 0,000 | 0,056 | 0,000 | 0,100 | 0,056 | 0,800 | 0,333 | 0,000 | 0,000 |
|  | **3** | 0,550 | 0,389 | 0,000 | 0,100 | 0,111 | 0,000 | 0,000 | 0,313 | 0,300 |
|  | **4** | 0,350 | 0,500 | 0,000 | 0,300 | 0,111 | 0,000 | 0,167 | 0,375 | 0,200 |
|  | **5** | 0,000 | 0,000 | 0,000 | 0,000 | 0,056 | 0,000 | 0,000 | 0,313 | 0,100 |
|  | **6** | 0,100 | 0,000 | 0,200 | 0,100 | 0,056 | 0,000 | 0,000 | 0,000 | 0,000 |
|  | **7** | 0,000 | 0,056 | 0,550 | 0,150 | 0,278 | 0,200 | 0,333 | 0,000 | 0,300 |
|  | **8** | 0,000 | 0,000 | 0,100 | 0,100 | 0,333 | 0,000 | 0,000 | 0,000 | 0,000 |
| **CnCirC7** | **N** |  |  |  |  |  |  |  |  |  |
|  | **1** | 0,778 | 0,750 | 0,063 | 0,056 | 0,438 | 0,000 | 0,500 | 0,929 | 0,056 |
|  | **2** | 0,222 | 0,188 | 0,313 | 0,333 | 0,313 | 0,563 | 0,375 | 0,000 | 0,389 |
|  | **3** | 0,000 | 0,000 | 0,250 | 0,278 | 0,125 | 0,000 | 0,000 | 0,000 | 0,111 |
|  | **4** | 0,000 | 0,063 | 0,375 | 0,333 | 0,125 | 0,438 | 0,125 | 0,071 | 0,444 |
| **CnCirE2** | **N** |  |  |  |  |  |  |  |  |  |
|  | **1** | 0,143 | 0,083 | 0,000 | 0,000 | 0,000 | 0,063 | 0,000 | 0,000 | 0,167 |
|  | **2** | 0,000 | 0,000 | 0,000 | 0,313 | 0,000 | 0,000 | 0,000 | 0,000 | 0,167 |
|  | **3** | 0,000 | 0,083 | 0,400 | 0,688 | 0,800 | 0,875 | 1,000 | 0,000 | 0,333 |
|  | **5** | 0,286 | 0,333 | 0,400 | 0,000 | 0,200 | 0,063 | 0,000 | 0,056 | 0,333 |
|  | **6** | 0,286 | 0,250 | 0,200 | 0,000 | 0,000 | 0,000 | 0,000 | 0,222 | 0,000 |
|  | **7** | 0,286 | 0,167 | 0,000 | 0,000 | 0,000 | 0,000 | 0,000 | 0,389 | 0,000 |
|  | **8** | 0,000 | 0,000 | 0,000 | 0,000 | 0,000 | 0,000 | 0,000 | 0,333 | 0,000 |
|  | **9** | 0,000 | 0,083 | 0,000 | 0,000 | 0,000 | 0,000 | 0,000 | 0,000 | 0,000 |
| **CnCirE10** | **N** |  |  |  |  |  |  |  |  |  |
|  | **1** | 0,000 | 0,000 | 0,056 | 0,000 | 0,000 | 0,111 | 0,000 | 0,000 | 0,313 |
|  | **2** | 0,000 | 0,000 | 0,111 | 0,167 | 0,222 | 0,056 | 0,000 | 0,111 | 0,063 |
|  | **3** | 0,125 | 0,000 | 0,111 | 0,000 | 0,222 | 0,056 | 0,188 | 0,000 | 0,000 |
|  | **4** | 0,625 | 0,222 | 0,611 | 0,667 | 0,333 | 0,778 | 0,563 | 0,111 | 0,500 |
|  | **5** | 0,250 | 0,778 | 0,111 | 0,167 | 0,222 | 0,000 | 0,250 | 0,778 | 0,125 |

| **Locus** | **Allele/n** | **BRTPF** | **BRTME** | **TONT** | **RTMT** | **MLT** | **RIT** | **VTT** | **WAT** | **PYT** |
| --- | --- | --- | --- | --- | --- | --- | --- | --- | --- | --- |
| **CnCirE12** | N |  |  |  |  |  |  |  |  |  |
|  | **1** | 0,071 | 0,278 | 0,278 | 0,167 | 0,222 | 0,150 | 0,167 | 0,000 | 0,350 |
|  | **2** | 0,214 | 0,000 | 0,000 | 0,000 | 0,000 | 0,000 | 0,000 | 0,000 | 0,000 |
|  | **3** | 0,357 | 0,611 | 0,389 | 0,333 | 0,222 | 0,350 | 0,500 | 0,050 | 0,150 |
|  | **4** | 0,357 | 0,111 | 0,000 | 0,000 | 0,111 | 0,000 | 0,056 | 0,050 | 0,000 |
|  | **5** | 0,000 | 0,000 | 0,278 | 0,000 | 0,000 | 0,000 | 0,056 | 0,450 | 0,100 |
|  | **6** | 0,000 | 0,000 | 0,000 | 0,167 | 0,111 | 0,200 | 0,056 | 0,050 | 0,250 |
|  | **7** | 0,000 | 0,000 | 0,000 | 0,000 | 0,111 | 0,000 | 0,056 | 0,050 | 0,000 |
|  | **8** | 0,000 | 0,000 | 0,056 | 0,333 | 0,222 | 0,300 | 0,111 | 0,350 | 0,150 |
| **CNZ40** | **N** |  |  |  |  |  |  |  |  |  |
|  | **1** | 0,000 | 0,000 | 0,000 | 0,450 | 0,000 | 0,000 | 0,071 | 0,000 | 0,200 |
|  | **2** | 0,100 | 0,000 | 0,583 | 0,250 | 0,056 | 0,222 | 0,071 | 0,000 | 0,700 |
|  | **3** | 0,100 | 0,000 | 0,167 | 0,000 | 0,111 | 0,000 | 0,357 | 0,000 | 0,000 |
|  | **4** | 0,150 | 0,500 | 0,000 | 0,100 | 0,278 | 0,111 | 0,071 | 0,250 | 0,000 |
|  | **5** | 0,650 | 0,350 | 0,167 | 0,200 | 0,444 | 0,667 | 0,429 | 0,688 | 0,100 |
|  | **6** | 0,000 | 0,150 | 0,083 | 0,000 | 0,000 | 0,000 | 0,000 | 0,063 | 0,000 |
|  | **8** | 0,000 | 0,000 | 0,000 | 0,000 | 0,111 | 0,000 | 0,000 | 0,000 | 0,000 |
| **CAC02** | **N** |  |  |  |  |  |  |  |  |  |
|  | **1** | 0,050 | 0,111 | 0,000 | 0,000 | 0,000 | 0,000 | 0,000 | 0,000 | 0,000 |
|  | **2** | 0,150 | 0,111 | 0,000 | 0,000 | 0,167 | 0,000 | 0,000 | 0,000 | 0,083 |
|  | **3** | 0,350 | 0,444 | 0,167 | 0,056 | 0,167 | 0,000 | 0,000 | 0,950 | 0,167 |
|  | **4** | 0,000 | 0,000 | 0,222 | 0,000 | 0,000 | 0,000 | 0,000 | 0,000 | 0,167 |
|  | **5** | 0,050 | 0,000 | 0,278 | 0,167 | 0,056 | 0,278 | 0,111 | 0,050 | 0,333 |
|  | **6** | 0,200 | 0,111 | 0,111 | 0,444 | 0,111 | 0,333 | 0,278 | 0,000 | 0,167 |
|  | **7** | 0,200 | 0,222 | 0,111 | 0,167 | 0,222 | 0,167 | 0,000 | 0,000 | 0,083 |
|  | **8** | 0,000 | 0,000 | 0,111 | 0,167 | 0,222 | 0,222 | 0,500 | 0,000 | 0,000 |
|  | **9** | 0,000 | 0,000 | 0,000 | 0,000 | 0,056 | 0,000 | 0,111 | 0,000 | 0,000 |
| **CNZ10** | **N** |  |  |  |  |  |  |  |  |  |
|  | **1** | 0,167 | 0,000 | 0,563 | 0,750 | 0,000 | 0,000 | 0,375 | 0,056 | 0,286 |
|  | **2** | 0,111 | 0,000 | 0,000 | 0,063 | 0,000 | 0,125 | 0,000 | 0,000 | 0,000 |
|  | **3** | 0,000 | 0,000 | 0,000 | 0,125 | 0,000 | 0,000 | 0,000 | 0,000 | 0,000 |
|  | **4** | 0,000 | 0,000 | 0,188 | 0,063 | 0,063 | 0,000 | 0,000 | 0,111 | 0,000 |
|  | **5** | 0,167 | 0,000 | 0,000 | 0,000 | 0,313 | 0,000 | 0,000 | 0,000 | 0,214 |
|  | **6** | 0,333 | 0,667 | 0,250 | 0,000 | 0,563 | 0,375 | 0,313 | 0,222 | 0,214 |
|  | **7** | 0,000 | 0,000 | 0,000 | 0,000 | 0,000 | 0,500 | 0,313 | 0,444 | 0,286 |
|  | **8** | 0,000 | 0,278 | 0,000 | 0,000 | 0,063 | 0,000 | 0,000 | 0,000 | 0,000 |
|  | **10** | 0,222 | 0,000 | 0,000 | 0,000 | 0,000 | 0,000 | 0,000 | 0,056 | 0,000 |
|  | **11** | 0,000 | 0,056 | 0,000 | 0,000 | 0,000 | 0,000 | 0,000 | 0,111 | 0,000 |

| **Locus** | **Allele/n** | **BRTPF** | **BRTME** | **TONT** | **RTMT** | **MLT** | **RIT** | **VTT** | **WAT** | **PYT** |
| --- | --- | --- | --- | --- | --- | --- | --- | --- | --- | --- |
| **CNZ43** | N |  |  |  |  |  |  |  |  |  |
|  | **1** | 0,000 | 0,200 | 0,000 | 0,000 | 0,000 | 0,000 | 0,000 | 0,000 | 0,000 |
|  | **2** | 0,375 | 0,550 | 0,000 | 0,000 | 0,125 | 0,000 | 0,000 | 0,000 | 0,000 |
|  | **3** | 0,188 | 0,000 | 0,000 | 0,000 | 0,000 | 0,167 | 0,700 | 0,056 | 0,071 |
|  | **4** | 0,000 | 0,000 | 0,500 | 0,125 | 0,000 | 0,000 | 0,000 | 0,000 | 0,000 |
|  | **5** | 0,000 | 0,200 | 0,100 | 0,125 | 0,375 | 0,000 | 0,000 | 0,722 | 0,143 |
|  | **6** | 0,063 | 0,000 | 0,400 | 0,625 | 0,500 | 0,000 | 0,000 | 0,000 | 0,429 |
|  | **7** | 0,375 | 0,000 | 0,000 | 0,125 | 0,000 | 0,833 | 0,100 | 0,000 | 0,357 |
|  | **8** | 0,000 | 0,050 | 0,000 | 0,000 | 0,000 | 0,000 | 0,000 | 0,167 | 0,000 |
|  | **9** | 0,000 | 0,000 | 0,000 | 0,000 | 0,000 | 0,000 | 0,200 | 0,056 | 0,000 |
| **CNZ44** | N |  |  |  |  |  |  |  |  |  |
|  | **1** | 0,000 | 0,000 | 0,300 | 0,000 | 0,000 | 0,000 | 0,000 | 0,000 | 0,000 |
|  | **2** | 0,000 | 0,000 | 0,200 | 0,000 | 0,000 | 0,000 | 0,000 | 0,000 | 0,450 |
|  | **3** | 0,000 | 0,000 | 0,000 | 0,000 | 0,300 | 0,000 | 0,000 | 0,750 | 0,050 |
|  | **4** | 0,000 | 0,000 | 0,150 | 0,000 | 0,300 | 0,000 | 0,150 | 0,250 | 0,000 |
|  | **5** | 0,000 | 0,000 | 0,250 | 0,500 | 0,200 | 0,556 | 0,050 | 0,000 | 0,300 |
|  | **6** | 0,167 | 0,444 | 0,100 | 0,500 | 0,200 | 0,444 | 0,600 | 0,000 | 0,200 |
|  | **7** | 0,333 | 0,111 | 0,000 | 0,000 | 0,000 | 0,000 | 0,200 | 0,000 | 0,000 |
|  | **8** | 0,500 | 0,444 | 0,000 | 0,000 | 0,000 | 0,000 | 0,000 | 0,000 | 0,000 |
| **CnCirB3** | N |  |  |  |  |  |  |  |  |  |
|  | **1** | 0,000 | 0,000 | 0,500 | 0,150 | 0,000 | 0,000 | 0,000 | 0,000 | 0,000 |
|  | **2** | 0,000 | 0,000 | 0,000 | 0,150 | 0,000 | 0,000 | 0,000 | 0,100 | 0,050 |
|  | **3** | 0,000 | 0,000 | 0,000 | 0,000 | 0,222 | 0,000 | 0,050 | 0,150 | 0,000 |
|  | **4** | 0,150 | 0,150 | 0,000 | 0,350 | 0,222 | 0,600 | 0,300 | 0,550 | 0,400 |
|  | **5** | 0,200 | 0,000 | 0,500 | 0,000 | 0,167 | 0,350 | 0,100 | 0,100 | 0,200 |
|  | **6** | 0,650 | 0,500 | 0,000 | 0,350 | 0,389 | 0,050 | 0,400 | 0,050 | 0,350 |
|  | **7** | 0,000 | 0,350 | 0,000 | 0,000 | 0,000 | 0,000 | 0,150 | 0,050 | 0,000 |
| **CNZ01** | N |  |  |  |  |  |  |  |  |  |
|  | **1** | 0,000 | 0,000 | 0,000 | 0,150 | 0,150 | 0,200 | 0,333 | 0,111 | 0,150 |
|  | **2** | 0,000 | 0,000 | 0,300 | 0,000 | 0,200 | 0,300 | 0,000 | 0,444 | 0,000 |
|  | **3** | 0,000 | 0,000 | 0,000 | 0,150 | 0,000 | 0,500 | 0,556 | 0,111 | 0,100 |
|  | **4** | 0,000 | 0,000 | 0,550 | 0,650 | 0,550 | 0,000 | 0,111 | 0,000 | 0,700 |
|  | **5** | 0,000 | 0,000 | 0,150 | 0,050 | 0,100 | 0,000 | 0,000 | 0,333 | 0,050 |
| **CNZ02** | N |  |  |  |  |  |  |  |  |  |
|  | **1** | 0,100 | 0,278 | 0,333 | 0,250 | 0,000 | 0,125 | 0,357 | 0,000 | 0,000 |
|  | **2** | 0,300 | 0,111 | 0,167 | 0,150 | 0,650 | 0,875 | 0,357 | 0,000 | 0,350 |
|  | **3** | 0,050 | 0,444 | 0,000 | 0,150 | 0,100 | 0,000 | 0,143 | 0,550 | 0,100 |
|  | **4** | 0,250 | 0,111 | 0,389 | 0,450 | 0,100 | 0,000 | 0,000 | 0,000 | 0,550 |
|  | **5** | 0,300 | 0,056 | 0,111 | 0,000 | 0,150 | 0,000 | 0,143 | 0,450 | 0,000 |

| **Locus** | **Allele/n** | **BRTPF** | **BRTME** | **TONT** | **RTMT** | **MLT** | **RIT** | **VTT** | **WAT** | **PYT** |
| --- | --- | --- | --- | --- | --- | --- | --- | --- | --- | --- |
| **CAC21** | N |  |  |  |  |  |  |  |  |  |
|  | **1** | 0,000 | 0,000 | 0,500 | 0,250 | 0,150 | 0,667 | 0,143 | 0,111 | 0,400 |
|  | **2** | 0,167 | 0,167 | 0,500 | 0,750 | 0,650 | 0,333 | 0,571 | 0,556 | 0,600 |
|  | **3** | 0,333 | 0,167 | 0,000 | 0,000 | 0,200 | 0,000 | 0,286 | 0,333 | 0,000 |
|  | **4** | 0,500 | 0,667 | 0,000 | 0,000 | 0,000 | 0,000 | 0,000 | 0,000 | 0,000 |
| **CAC23** | N |  |  |  |  |  |  |  |  |  |
|  | **1** | 0,000 | 0,150 | 0,000 | 0,000 | 0,000 | 0,000 | 0,000 | 0,000 | 0,250 |
|  | **2** | 1,000 | 0,700 | 0,600 | 0,200 | 0,167 | 0,000 | 0,333 | 0,000 | 0,300 |
|  | **3** | 0,000 | 0,150 | 0,200 | 0,200 | 0,611 | 1,000 | 0,611 | 0,400 | 0,400 |
|  | **4** | 0,000 | 0,000 | 0,200 | 0,600 | 0,222 | 0,000 | 0,056 | 0,600 | 0,050 |
| **CAC71** | N |  |  |  |  |  |  |  |  |  |
|  | **1** | 0,000 | 0,000 | 0,050 | 0,000 | 0,000 | 0,000 | 0,000 | 0,000 | 0,188 |
|  | **2** | 0,000 | 0,000 | 0,250 | 0,444 | 0,000 | 0,944 | 0,250 | 0,250 | 0,563 |
|  | **3** | 0,000 | 0,000 | 0,700 | 0,556 | 0,944 | 0,056 | 0,688 | 0,667 | 0,250 |
|  | **4** | 0,000 | 0,000 | 0,000 | 0,000 | 0,056 | 0,000 | 0,063 | 0,083 | 0,000 |
| **CAC84** | N |  |  |  |  |  |  |  |  |  |
|  | **2** | 0,050 | 0,056 | 0,000 | 0,000 | 0,000 | 0,000 | 0,000 | 0,063 | 0,000 |
|  | **3** | 0,300 | 0,111 | 0,000 | 0,000 | 0,000 | 0,000 | 0,000 | 0,000 | 0,000 |
|  | **4** | 0,000 | 0,000 | 0,917 | 0,611 | 0,889 | 1,000 | 1,000 | 0,000 | 0,833 |
|  | **5** | 0,300 | 0,000 | 0,000 | 0,222 | 0,111 | 0,000 | 0,000 | 0,938 | 0,000 |
|  | **6** | 0,350 | 0,833 | 0,083 | 0,167 | 0,000 | 0,000 | 0,000 | 0,000 | 0,167 |
| **CAC50** | N |  |  |  |  |  |  |  |  |  |
|  | **1** | 0,000 | 0,000 | 0,000 | 0,000 | 0,278 | 0,300 | 0,500 | 0,000 | 0,333 |
|  | **2** | 0,000 | 0,000 | 0,250 | 0,000 | 0,278 | 0,600 | 0,500 | 0,000 | 0,167 |
|  | **3** | 0,000 | 0,000 | 0,100 | 0,375 | 0,000 | 0,000 | 0,000 | 0,250 | 0,333 |
|  | **4** | 0,050 | 0,278 | 0,250 | 0,000 | 0,111 | 0,100 | 0,000 | 0,550 | 0,000 |
|  | **5** | 0,450 | 0,333 | 0,350 | 0,250 | 0,333 | 0,000 | 0,000 | 0,000 | 0,167 |
|  | **6** | 0,000 | 0,000 | 0,000 | 0,000 | 0,000 | 0,000 | 0,000 | 0,100 | 0,000 |
|  | **7** | 0,500 | 0,389 | 0,050 | 0,375 | 0,000 | 0,000 | 0,000 | 0,100 | 0,000 |
|  |  |  |  |  |  |  |  |  |  |  |
|  |  |  |  |  |  |  |  |  |  |  |
|  |  |  |  |  |  |  |  |  |  |  |
|  |  |  |  |  |  |  |  |  |  |  |
|  |  |  |  |  |  |  |  |  |  |  |
|  |  |  |  |  |  |  |  |  |  |  |
|  |  |  |  |  |  |  |  |  |  |  |
|  |  |  |  |  |  |  |  |  |  |  |
|  |  |  |  |  |  |  |  |  |  |  |
|  |  |  |  |  |  |  |  |  |  |  |
